# Dispersed-Monolayer Graphene-Doped Polymer/Silica Hybrid Mach-Zehnder interferometer (MZI) Thermal Optical Switch with Low-Power Consumption and Fast Response

**DOI:** 10.3390/polym11111898

**Published:** 2019-11-18

**Authors:** Yue Cao, Daming Zhang, Yue Yang, Baizhu Lin, Jiawen Lv, Xianwang Yang, Haowen Zhao, Fei Wang, Baohua Li, Yunji Yi

**Affiliations:** State Key Laboratory of Integrated Optoelectronics, College of Electronic Science and Engineering, Jilin University, Changchun 130012, China; yuecao17@mails.jlu.edu.cn (Y.C.); zhangdm@jlu.edu.cn (D.Z.); a2604702999@163.com (Y.Y.); linbz17@mails.jlu.edu.cn (B.L.); lvjw18@mails.jlu.edu.cn (J.L.); yangxw1918@mails.jlu.edu.cn (X.Y.); zhaohw1918@mails.jlu.edu.cn (H.Z.); wang_fei@jlu.edu.cn (F.W.); libh@jlu.edu.cn (B.L.)

**Keywords:** thermal optical switch, dispersed-monolayer graphene, hybrid integrated waveguide, thermal conductivity, Lewis–Nielsen model

## Abstract

This article demonstrates a dispersed-monolayer graphene-doped polymer/silica hybrid Mach–Zehnder interferometer (MZI) thermal optical switch with low-power consumption and fast response. The polymer/silica hybrid MZI structure reduces the power consumption of the device as a result of the large thermal optical coefficient of the polymer material. To further decrease the response time of the thermal optical switch device, a polymethyl methacrylate, doped with monolayer graphene as a cladding material, has been synthesized. Our study theoretically analyzed the thermal conductivity of composites using the Lewis–Nielsen model. The predicted thermal conductivity of the composites increased by 133.16% at a graphene volume fraction of 0.263 vol %, due to the large thermal conductivity of graphene. Measurements taken of the fabricated thermal optical switch exhibited a power consumption of 7.68 mW, a rise time of 40 μs, and a fall time of 80 μs at a wavelength of 1550 nm.

## 1. Introduction

High-speed optical switch chips are core components in optical switching systems, meeting the requirements of being high speed, large capacity, and low delay [1,2,3]. Optical switches include conventional mechanical optical switches [4], waveguide switches such as electro-optical switches [5], acoustic-optical switches [6], and thermal optical (TO) switches. TO switch devices have attracted attention recently, due to their uniquely compact structure, low production cost, ease of integration, and good stability [7,8,9].

According to the structure of the device, TO switches can be categorized into X-junction TO switches [10] and Mach–Zehnder interferometer (MZI) switches. In 2000, N. Keil fabricated an X-junction polymer/silica thermo-optic switch with a power of less than 80 mW [11]. In 2019, Y. Gao demonstrated a polymer/silica MZI thermo-optic switch as having a power consumption of 15.4 mW at 1550 nm, and a rise time and fall time of 71.5 and 140.3 μs, respectively [12]. Compared with X-junction TO switches, the MZI TO switches display both lower power consumption and faster response times. According to previous literature, TO switches can be categorized into inorganic TO switches, polymer TO switches, and polymer/inorganic hybrid TO switches [12,13,14]. In 2003, Espinola R. fabricated MZI thermo-optic switches on thin silicon-on-insulator (SOI) substrates, with a switching power of 50 mW and a rise time of less than 3.5 μs [8]. In 2017, Sun S. demonstrated an all-polymer MZI switch, which presented a low power consumption of 2.8 mW and a response time of 600.0 μs [15]. In 2018, Q. Xu fabricated an MZI thermal optical switch based on the organic–inorganic hybrid DR1/SiO_2_−TiO_2_ waveguide [14]. A switching power of 8.9 mW and response times of 80 μs were obtained at a wavelength of 1550 nm. Because of the large thermal conductivity of inorganic material and the large thermal optical coefficient of the polymer, the response time of polymer/silica hybrid TO switches is faster than that of polymer TO switches. To realize a faster response time and lower power consumption for the polymer/silica hybrid MZI TO switch, the graphene-based TO switch has been studied, due to the large thermal conductivity of graphene [16,17,18]. However, with the graphene film-assisted TO switch, the graphene film is uneven and fragile during the graphene transfer and electrode fabrication processes.

In this research, to solve the problems of the graphene film being uneven and fragile during the fabrication of the TO switch, a graphene-dispersed N-Methyl pyrrolidone (NMP) material was synthesized and doped into polymethyl methacrylate (PMMA). This composite material not only guaranteed the large thermal optical coefficient of the polymer, but also an improved thermal conductivity, due to the large thermal conductivity of graphene [18]. Therefore, as a result of the doping of dispersed-monolayer graphene into the cladding material, the proposed hybrid TO switch should display both low-power consumption and fast response characteristics. Moreover, the doped graphene material is not sensitive to the absorption of various polarized modes, which solves the limitation of polarization dependence with waveguide modes. 

## 2. Materials and Methods 

### 2.1. Thermal Conductivity of Graphene-Doped PMMA Material

The thermal conductivity of the material significantly affects the performance of TO switches. The thermal conductivity of the composite material has been analyzed and calculated using the Bruggeman [19] and Lewis–Nielsen models [20,21], which are generally used to predict the thermal conductivity of a composite material. In the Bruggeman model, the thermal conductivity of composites can be calculated under the condition of having a low filler-volume fraction, which can be calculated by:

(1) $$1-V=\frac{{Κ}_{g}-{Κ}_{c}}{{Κ}_{g}-{Κ}_{p}}{\left(\frac{{Κ}_{p}}{{Κ}_{c}}\right)}^{\frac{1}{3}}$$

where *K*_p_ and *K*_g_ are the thermal conductivities of the PMMA matrix and graphene filler [18,22], respectively (*K*_p_ = 0.19 W/mk; *K*_g_ = 5300 W/mk). However, the limitation of the model is that the interfacial thermal resistance between the fillers and the polymer is not considered.

In the Lewis–Nielsen model, the effects of particle morphology, aggregation method, and orientation on the thermal conductivity of the composites are considered, and the thermal conductivity is calculated by the expression:

(2) $$K_{c}=K_{p}\frac{1+ABV}{1-B\Psi V}$$

where

(3) $$A=\sqrt{3}\log\sqrt{\frac{D}{L}}$$

(4) $$B=\frac{{Κ}_{g}K_{p}-1}{K_{g}/K_{p}+A}$$

(5) $$\Psi =1+\frac{V(1-V_{m})}{V_{m}^{2}}$$

where *D* and *L* represent the diameter and thickness of the filler, respectively (*D* = 1 μm; *L* = 3 nm). *A* and *B* depend on the filler dimensions and the thermal conductivity of each component, respectively, and *V* is the volume fraction of filler. *V*_m_ is the maximum packing fraction of the filler (*V*_m_ = 0.673).

**Figure 1:**
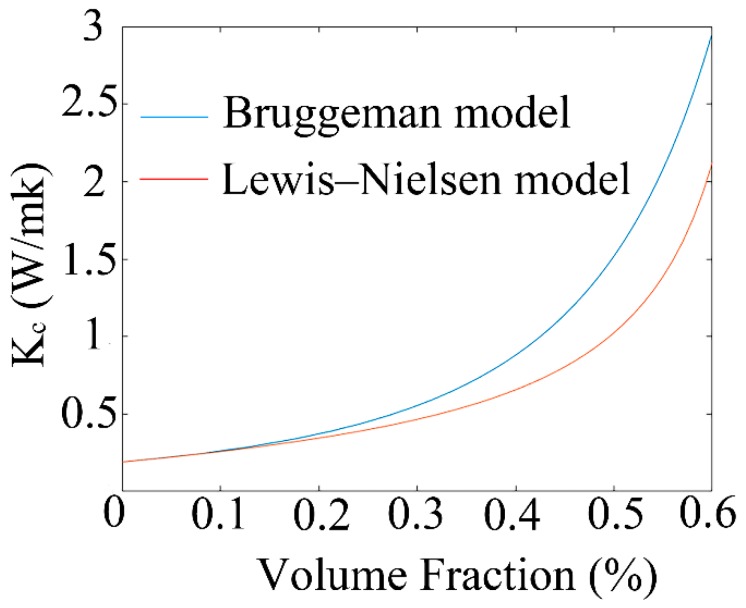
Calculated thermal conductivity of composites with different volume fractions of graphene filler.

The thermal conductivity of composites as a function of the volume fraction of filler, calculated using MATLAB software (MathWorks, Natick, MA, USA), is shown in Figure 1. The blue and red lines represent the composites’ thermal conductivities as calculated by the Bruggeman and Lewis–Nielsen models, respectively. For the low graphene doping region, the calculated results of the two models are similar. In contrast, the calculation deviation of the Bruggeman model is relatively large for the high doping ratios of the monolayer graphene material. Compared with the thermal conductivity of the PMMA material, the calculated thermal conductivity of the composites is increased by 133.16% when doping the dispersed-monolayer graphene at 0.263 vol % into the PMMA material.

### 2.2. Application of Composite Material in TO Switch Field

#### 2.2.1. Device Structure and Light Field Distributions

**Figure 2:**
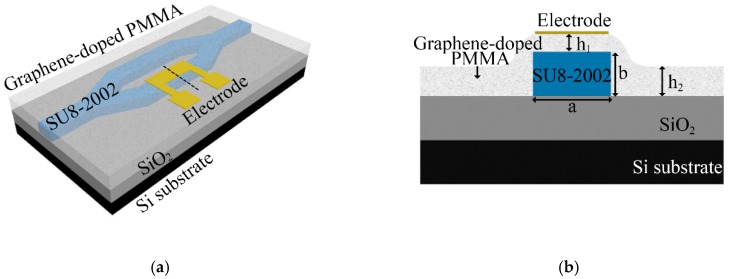
The arc cladding structure of the graphene-doped thermal optical (TO) switch in (**a**) a three-dimensional direction and (**b)** a cross-sectional direction.

**Figure 3:**
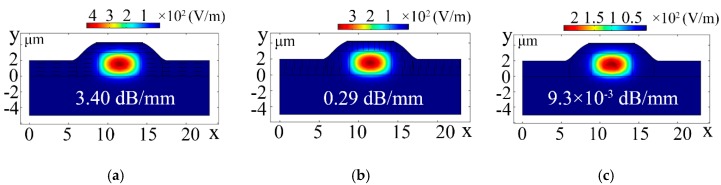
Light field distributions of the dispersed-monolayer graphene arrangement in the (**a**) *x*-direction and (**b**) *y*-direction, and the (**c**) ND-TO switch.

Figure 2 shows the arc cladding structure of the polymer/silica hybrid MZI TO switch doped with the dispersed-monolayer graphene NMP material (GD-TO switch). The thickness and width of the core layer were *a* = 5.3 μm and *b* = 3 μm, respectively. The distance between the electrode and the top of the core was *h*_1_ = 1.2 μm, and the thickness of the cladding was *h*_2_ = 2 μm. The refractive indexes of the imaginary and real parts of the graphene material are 2.79 and 2.97, respectively [23,24]. The refractive index of PMMA is 1.495 [25]. Figure 3 shows the light field distributions of the arc cladding structure of the TO switch. The light absorption of monolayer graphene for transverse-electric (TE) modes is considered with transverse-magnetic (TM) modes because the arrangement of the doped graphene material in the polymer is random. The effective refractive index and the loss of TE modes were calculated using finite element analysis software when all the graphene was arranged in a horizontal direction or in a longitudinal direction. The TE mode insertion losses of the dispersed-monolayer graphene arrangement in the x-direction and y-direction were 3.40 and 0.29 dB/mm, respectively. The loss of the non-doped graphene TO switch (ND-TO switch) was 0.0093 dB/mm. In order to counteract the absorption loss from the doped monolayer graphene, the structure of the device can be further optimized, by using an embedded waveguide structure with a low refractive index layer [23,24,26]. Alternatively, the length of the thermo-optic device can be reduced to decrease absorption loss.

#### 2.2.2. Device Performance Simulation

**Figure 4:**
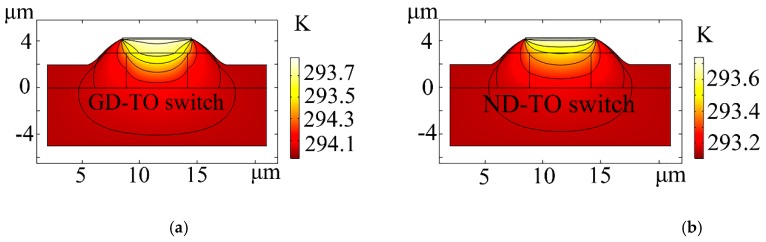

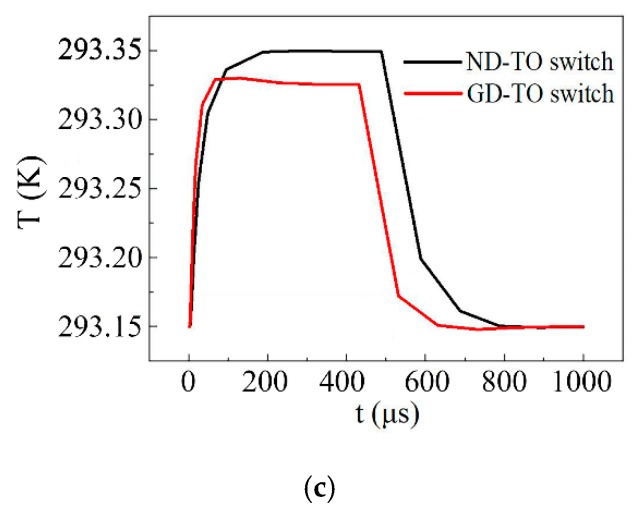
(**a**) and (**b**) thermal field distributions; and (**c**) calculated response times for the graphene doped (GD)-TO switch (*Rise time* = 30 μs; *Fall time* = 110 μs), and non-doped graphene (ND)-TO switch (*Rise time* = 78 μs; *Fall time* = 164 μs).

The calculated response times and the thermal field distribution of the arc cladding structure for the GD-TO and ND-TO switches are shown in Figure 4. When heating the electrode, the refractive index of the polymer decreases, while the refractive index of the silica increases, realizing the phase shift ∆Φ of π. The initial temperature is set at 293.15 K. The calculated power consumptions, when the phase shift ∆Φ was π [27], were 3.24 and 3.79 mW, respectively. Furthermore, the rise time and fall time of the GD-TO switch decreased by 61.54% and 32.93%, respectively. Compared with the traditional, non-doped hybrid TO switch, the proposed device has both a significantly wider heat distribution and a lower power consumption. The calculated response time is significantly reduced, due to the large thermal conductivity of the composite material.

## 3. Results

### 3.1. Fabrication and Characterizations 

**Figure 5:**
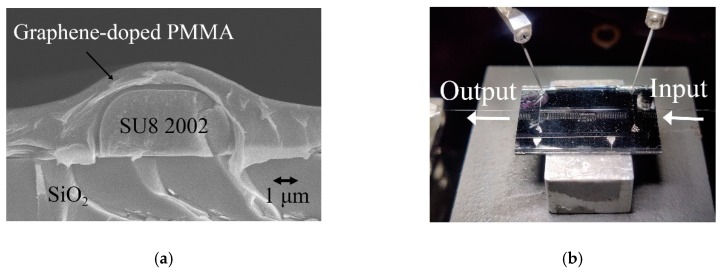
(**a**) The scanning electron microscope image, and (**b**) the photograph of the test system of the graphene-based TO switch.

Following the previous simulation, the polymer/silica hybrid MZI TO switch was fabricated using doped dispersed-monolayer graphene NMP material (Tanfeng Tech.Inc, Suzhou, China). Figure 5 shows a scanning electron microscope (SEM) image (JEOL, Munich, Germany) and a photograph of the device test system (SURUGA SEIKI CO., LTD., Tokyo, Japan). The refractive indexes of the core layer and the cladding material were 1.572 and 1.48, respectively, at a wavelength of 1550 nm. The device fabrication processes were as follows: firstly, a 3 μm thick core layer was fabricated on SiO_2_, using spin-coating and photolithography processes (ABM-USA, Inc., San Jose, CA, USA). The width of the core was 5.3 μm. Next, a graphene-dispersed NMP material was synthesized and doped with 0.263 vol % into PMMA (Tokyo chemical industry CO., LTD., Tokyo, Japan). The composites were spin-coated on the core layer as a waveguide cladding. The arc cladding structure was realized by optimizing the viscosity of the cladding material and the spin rate of the spin coater (Laurell, Inc., North Wales, PA, USA). Lastly, a 0.1 μm thick Al electrode was fabricated with a width and length of 6 μm and 1.5 cm, respectively. According to the Lewis–Nielsen model, the calculated thermal conductivity of the composites was increased by 133.16%.

### 3.2. Power Consumption and Response Time 

**Figure 6:**
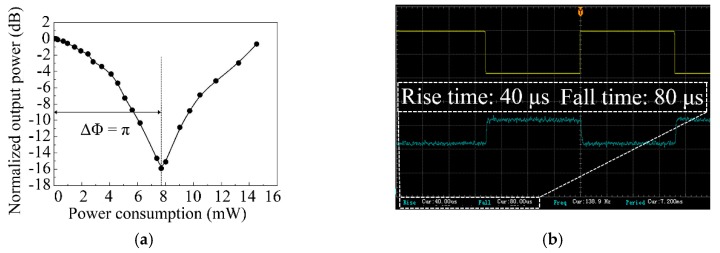
(**a**) Measured output power of the graphene-doped TO switch with different driving powers at a wavelength of 1550 nm, and (**b**) the response waveforms measured by an oscilloscope (**upper trace**: input electric signal; **lower trace**: output optical power signal).

Figure 6a shows the relationship between the normalized output power of the graphene-doped TO switch and its power consumption at a wavelength of 1550 nm. The measured insertion loss was 31.13 dB, realizing the ON state of the TO switch. The measured extinction ratio was 15.88 dB at a power consumption of 7.68 mW, realizing the OFF state of the device. Figure 6b shows the response times of the TO switch, measured by an oscilloscope (Beijing RIGOL Technology Co., Ltd. Beijing, China). When applying a square wave voltage of 138.9 Hz, the rise and fall times were 40 and 80 μs, respectively. The device exhibited a fast response speed, due to the improved thermal conductivity of the cladding material. 

## 4. Conclusions

In conclusion, we proposed a polymer/silica hybrid MZI TO switch. The predicted thermal conductivity of the composites was then increased by 133.16% for the composites doped with graphene (0.263 vol %). The fabricated TO switch exhibited a power consumption of 7.68 mW, a rise time of 40 μs, and a fall time of 80 μs at a wavelength of 1550 nm. The doped graphene material was not sensitive to the absorption of various polarized modes. In future research, the application of graphene in the all-optical switch field will be studied according to the photothermal efficiency characteristics of graphene at different wavelengths. Moreover, graphene-doped materials have potential applications in both flexible thermal optical switches and wearable fields.
